# A Rosetta Stone for Breast Cancer: Prognostic Value and Dynamic Regulation of Neutrophil in Tumor Microenvironment

**DOI:** 10.3389/fimmu.2020.01779

**Published:** 2020-08-07

**Authors:** Wei Zhang, Yimin Shen, Huanhuan Huang, Sheng Pan, Jingxin Jiang, Wuzhen Chen, Ting Zhang, Chao Zhang, Chao Ni

**Affiliations:** ^1^Department of Endocrinology, Zhejiang Provincial People's Hospital, People's Hospital of Hangzhou Medical College, Hangzhou, China; ^2^Department of Cardiology, Second Affiliated Hospital, Zhejiang University, Hangzhou, China; ^3^Key Laboratory of Tumour Microenvironment and Immune Therapy of Zhejiang Province, Second Affiliated Hospital, Zhejiang University, Hangzhou, China; ^4^Department of Breast Surgery, Second Affiliated Hospital, Zhejiang University, Hangzhou, China; ^5^School of Medicine, Chu Kochen Honors College, Zhejiang University, Hangzhou, China; ^6^Department of Anatomy, School of Medicine, Zhejiang University, Hangzhou, China

**Keywords:** breast cancer, immuno-therapy, neutrophils, neutrophil-to-lymphocyte ratio, tumor microenvironment

## Abstract

Increasing evidence has revealed that the initiation and progression of breast cancer are greatly affected by the immune environment. Neutrophils are the most abundant leucocytes in circulation and act as the spearhead in inflammation, including in breast cancer. Circulating neutrophils are closely related to the prognosis of breast cancer patients, and tumor-infiltrating neutrophils have varied functions at different stages of breast cancer, such as antitumor or tumor-promoting neutrophils, which are termed N1 and N2 neutrophils, respectively. In this review, we will discuss the utility of circulating neutrophils for predicting prognosis and therapeutic efficacy and the underlying mechanisms of their chemotaxis, the dynamic regulation of their antitumor or protumor functions and their different spatial distributions in tumor microenvironment. Finally, we also discuss the possibility of targeting neutrophils as a therapeutic strategy in breast cancer.

## Introduction

Breast cancer (BC) is the most common malignancy in women worldwide (1). Although BC is classified as a malignant disease with low immunogenicity, recent evidence has revealed a promising outcome of therapies with blocking immune checkpoints in both early and advanced stages (2–4). The efficacy of immunotherapy is closely related to the tumor immune microenvironment, especially to infiltrating immune cells (5). To date, macrophages and T cells are the most well-studied immune cells in BC, whereas increasing evidence has indicated that neutrophils are also key in the oncogenesis and metastasis of BC; in addition, circulating neutrophils have been reported to have great prognostic prediction value (6). Neutrophils are the most abundant leucocytes in blood and usually act as the first line of host defense against pathogens (7). However, due to their short life span (an average of 6–8 h in blood) (8), it is difficult to employ this subset of cells for experiments, which has resulted in a poor understanding of their role in solid tumors. In addition, some contradictory results reported *in vitro* studies or animal experiments have suggested a dual effect of neutrophils in tumor development.

Neutrophils can present both antitumorigenic (“N1”) and protumorigenic (“N2”) phenotypes in various cancers or specific circumstances. The term neutrophil in several studies also includes both mature neutrophils and myeloid-derived suppressor cells (MDSCs). MDSCs are described as a subset of neutrophils with immunosuppressive functions that express CD11b and Gr1 (9, 10) and can be divided into monocytic (M) (CD11b+/Ly6C+) MDSCs and G/PMN (CD11b+/Ly6G+) MDSCs (11), and G/PMN MDSCs usually share a common set of markers and similar morphological features with neutrophils (9).

To avoid confusion, we mainly focus on the biological function of mature neutrophils and related therapeutic strategies for targeting them in BC. We provide a comprehensive review of the prognostic value of circulating neutrophils and the mechanisms of how tumor-associated neutrophils (TANs) exert antitumor or tumor-promoting functions in BC, and in the end, we also discuss the potential of targeting neutrophils as a therapeutic strategy in cancer.

## Prognostic Value of the Neutrophil-to-Lymphocyte Ratio (NLR)

Tumors can be thought of as wounds that will not heal and are characterized by chronic inflammation. Neutrophils are the most rapidly responding immune cells to inflammation, and many studies have found that the NLR is closely related to the prognosis and treatment response in patients bearing BC (12, 13). A recent meta-analysis of 39 studies, including 17,079 patients with both early and advanced BC, revealed that patients with a higher NLR before treatment had poorer disease-free survival (DFS) than those with a lower NLR before treatment, but the NLR was not related to overall survival (OS); the subgroup analysis found that the NLR was associated with prognosis only in early-stage patients but not in patients with metastasis (14). Since similar meta-analyses were not based on individual patient data, which may cause significant bias, we reviewed and compared the individual reports and found some issues worth discussing here. Widmann et al. first reported the correlation between the NLR and BC prognosis in 316 patients, and it was found that a higher NLR (≥3.3) before treatment was an adverse factor for both short- and long-term mortality (15). The majority of retrospective studies thereafter have drawn similar conclusions (16–19), and the NLR was found to be consistent among different BC subtypes at baseline (20, 21). However, a prospective substudy of GEICAM/9906, which comprised 1,246 patients, did not find any prognostic value of the NLR after adjustment for clinicopathological factors; in addition, a high NLR was independently associated with worse DFS in only high-risk patients (the hormone receptor-negative/HER2+ population and in patients with ≥3 lymph node metastases) (22). Another study with 247 early BC patients also found that the NLR before surgery was not associated with DFS (23), indicating that the presurgery NLR may be valuable only in patients with a high tumor burden.

In addition to the above studies, several studies also explored the prognostic value of the NLR posttreatment or with continuous assessment. A retrospective study comparing the absolute lymphocyte count (ALC) and the NLR eight consecutive times before and after chemotherapy found that patients who died had lower ALC and higher NLR values than patients who remained alive throughout the treatment course; additionally, among the patients who died, a steady increase in the NLR over the baseline measurement was observed at subsequent time points (24). Another retrospective study included 330 BC patients with DFS values of more than 5 years, and it interestingly found that NLR sampled during follow-up rather than before any treatment was an independent prognostic factor for late recurrence (21). However, there is still no compelling explanation for the abovementioned inconsistent results. In addition, since lymphocytes are critical in cancer immune surveillance and neutrophils have been reported to play a protumor role in most studies, low lymphocytes and high neutrophils in circulation may also suggest immunosuppression status (10), and studies focused on the relationship between neoadjuvant chemotherapy (NCT) and the NLR might support the above hypothesis. A comprehensive review of the existing reports shows that most studies have found that a low NLR indicates a higher NCT response and pathological complete response (pCR) rate (25–27); in addition, the NLR has showed predictive value not only in all molecular types of BC but also in both operable and locally advanced BC (18, 28, 29). Interestingly, although Suppan et al. did not find a significant correlation between the initial NLR and prognosis, the same cohort revealed a low NLR as a significant parameter for predicting chemotherapy response (*p* = 0.012) (23). A low NLR was also reported to be associated with a higher response rate to primary endocrine therapy for locally advanced or metastatic BC (30, 31).

Although increasing evidence suggests a close association between the NLR and prognosis in BC, several issues remain that make clinical application difficult. One of the most important reasons is the lack of a consensus cut-off value. As we list here (Table 1), the cut-off values for the NLR in the published studies were between 2 and 4. In addition, based on individual studies, the sensitivity of the NLR fluctuates greatly (50–94.1%), and the specificity is much lower (26.5–51.6%) (18, 29, 49). Therefore, some researchers have tried to determine a better alternative parameter. In addition to the NLR, the platelet-to-lymphocyte (PLR) ratio has also been investigated and compared with the NLR in BC. A single central retrospective study with 434 hormone receptor-negative non-metastatic BC patients reported that both elevated NLR and PLR were associated with poor OS; however, the multivariate analysis revealed that only the NLR (*p* < 0.001) but not the PLR (*p* = 0.104) was a significant indicator for both DFS and OS (50). Additionally, since the absolute lymphocyte count has also been reported as a prognostic factor, the predictive values of the PLR and NLR were evaluated after adjusting for the total lymphocyte count. The results showed that the PLR was no longer a significant predictor for 5-year mortality, and the NLR remained a significant predictor irrespective of the lymphocyte count (51). Furthermore, it was revealed that the combination of the NLR and PLR could further improve the predictive value. Two retrospective studies found that the highest rate of pCR (32%) was in the group of patients with an NLR^low^/PLR^low^ profile, and the lowest rate (19%) was in the group with an NLR^high^/PLR^high^ profile (18); in addition, when the cut-off values for the NLR and PLR were applied, the specificity of predicting a pCR increased from 38 to 52% (49).

**Table 1:** Characteristics of the studies related to neutrophil-to-lymphocyte ratio.

**References**	**Country**	**Study period**	**Cancer type**	**Median age (ys)**	**No. patients low/high NLR**	**Treatment**	**Follow-up**	**Significance of NLR**
Noh et al. (32)	Korea	2000–2010	Luminal A/B, HER2-enriched, TNBC	50	*n* = 442 NLR < 2.5 (*n* ≤ 327) NLR ≥ 2.5 (*n* = 115)	NR	5.9 ys	High NLR indicates lower survival rate (*p* = 0.009).
Koh et al. (33)	Korea	2002–2010	ER/PR-positive, HER2-enriched	44	*n* = 157 NLR ≤ 2.25 (*n* = 91) NLR > 2.25 (*n* = 66)	Surgery, NCT	21 mo	Univariate analysis indicates high NLR related to lower RFS (*p* = 0.001) and OS (*p* < 0.001).
Yao et al. (34)	China	2009–2011	Luminal A/B, ER/PR-positive, HER2-enriched, TNBC	50	*n* = 608 NLR = 2.57; NLR > 2.57	Surgery	5.9 ys	High NLR indicates lower 5-year OS.
Pistelli et al. (35)	Italy	2006–2012	TNBC	53	*n* = 90 NLR ≤ 3 (*n* = 73) NLR > 3 (*n* = 17)	NR	53.8 mo	Multivariate analysis indicates high pretreatment NLR is correlated with poor DFS (*p* = 0.03) and OS (*p* = 0.01).
Ulas et al. (36)	Turkey	2009–2014	HER2-enriched	51.4	*n* = 187 NLR < 2.38 (*n* = 119) NLR > 2.38 (*n* = 68)	Adjuvant transtuzumab	26 mo	High pretreatment NLR indicates shorter DFS.
Jia et al. (37)	China	2000–2010	ER /PR-positive, HER2-enriched, TNBC	47	*n* = 1,570 NLR > 2 (*n* = 804) NLR ≤ 2 (*n* = 766)	NCT, surgery	79 mo	Multivariate analysis indicates low NLR is related to superior DFS (*p* = 0.004) and (*p* = 0.022).
Bozkurt et al. (38)	Turkey	2002–2013	TNBC	50	*n* = 85 NLR ≤ 2 (*n* = 33) NLR > 2 (*n* = 52)	Surgery, adjuvant chemotherapy, and radiotherapy	60 mo	Multivariate analysis indicates high pretreatment NLR is correlated with poor DFS (*p* = 0.006) and OS (*p* = 0.04).
Asano et al. (25)	Japan	2007–2013	TNBC	56	*n* = 177 NLR < 3 (*n* = 58) NLR > 3 (*n* = 119)	NCT	3.4 ys	Univariate analysis indicates low NLR is related to favorable prognosis in TNBC patients who achieved pCR (*p* = 0.044, hazard ratio = 0.06).
Rimando et al. (39)	USA	2001–2013	Non-metastatic BC	58	*n* = 461 NLR ≤ 3.7 (*n* = 409) NLR > 3.7 (*n* = 52)	Radiotherapy, chemotherapy	61 mo	High pretreatment NLR indicates poor all-cause mortality, with a multivariable HR of 2.31 (95% CI: 1.10–4.86).
Iwase et al. (40)	Japan	2005–2014	TNBC	50.9	*n* = 89 NRL = 3	Chemotherapy	NR	High NLR upon recurrence indicates shorter OS recurrence rates (*p* < 0.05).
Hernandez et al. (41)	Spain	2003–2016	Luminal A/B, ER/PR-positive, HER2-enriched, TNBC	49.8	*n* = 150 NLR = 3.3	NCT, surgery	24 mo	Low NLR indicates higher OS (*p* = 0.024).
Miyagawa et al. (42)	Japan	2010–2017	Locally Advanced or Metastatic BC	63	*n* = 59 NLR < 3 (*n* = 24) NLR ≥ 3 (*n* = 35)	Eribulin	NR	Low NLR indicates better PFS (*p* = 0.0032).
Ferroni et al. (43)	Italy	2007–2017	Luminal A/B, HER2-enriched, TNBC	57	*n* = 475 NLR ≤ 2 (*n* = 245) NLR > 2 (*n* = 230)	NCT, chemotherapy, endocrine therapy; trastuzumab regimens	45.6 mo	High pretreatment NLR indicates worse DFS (HR = 2.28) and OS (HR = 3.39).
Qiu et al. (44)	China	2006–2013	Non-metastatic TNBC	50	*n* = 406 NLR < 2.85 (*n* = 210) NLR ≥ 2.85 (*n* = 196)	Surgery, NCT, chemotherapy	54.3 mo	Low NLR indicates higher OS (*p* < 0.001) and DFS (*p* < 0.001).
Iimori et al. (30)	Japan	2004–2013	Luminal A/B, HER2-enriched, TNBC	63	*n* = 34 NLR < 3 (*n* = 24) NLR ≥ 3 (*n* = 10)	Endocrine therapy	38.8 mo	Low NLR indicates a prolongation of PFS (*p* = 0.003) and OS (*p* = 0.013).
Mando et al. (45)	Argentina	2011–2014	Early stage BC	56	*n* = 85 NRL = 2	Surgery	38.6 mo	High NLR indicates lower DFS (*p* = 0.048).
Lee et al., (46)	Korea	2008–2015	TNBC	51	*n* = 358 NLR ≤ 3.16 (*n* = 313) NLR > 3.16 (*n* = 45)	NCT	NR	Low NLR indicates superior OS (*p* = 0.002) and DFS (*p* = 0.032).
Xuan et al. (19)	China	2006–2008	TNBC	50	*n* = 286 NLR < 2.93 (*n* = 223) NLR ≥ 2.93(*n* = 63)	Surgery	NR	Low NLR indicates longer DFS (*p* = 0).
Fujmoto et al. (47)	Japan	2005–2016	With high counts of lymphocytes	30.7	*n* = 889 NLR < 2.72 (*n* = 582) NLR >2.72 (*n* = 307)	Surgery, adjuvant chemotherapies, endocrine therapies	NR	Low NLR indicates better RFS (*p* = 0.036).
Imamura et al. (48)	Japan	2011–2017	HER2-enriched	53	*n* = 53 NLR < 2.56 (*n* = 26) NLR ≥ 2.56 (*n* = 27)	Trastuzumab emtansine	NR	Low NLR at baseline indicates better PFS (*p* = 0.0001) and OS (*p* = 0.0296).

*NLR, Neutrophil-to-lymphocyte ratio; ER, Estrogen receptor; PR, Progesterone receptor; HER2, Human epidermal growth factor receptor 2; Mo, Months; Ys, Years; DFS, Disease-free survival; OS, Overall survival; PFS, Progression-free survival; RFS, Relapse free survival; pCR, Pathological complete response; TNBC, Triple-negative breast cancer; NCT, Neoadjuvant chemotherapy; NR, Not recorded*.

However, the causal relationship between the NLR and poor prognosis in malignant disease has yet to be illuminated. According to an assessment with paired peripheral blood and pancreatic cancer specimens, Takakura et al. found that a high NLR was associated with increased tumor-associated macrophages (TAMs) and decreased tumor-associated lymphocytes but was not significantly related to CD66b+ infiltrating neutrophils (52). Therefore, it seems that an increase in neutrophils in peripheral blood is not necessarily related to the number of TANs. Several basic studies have suggested a unique mechanism of the pro-tumor function of circulating neutrophils: protecting circulating tumor cells (CTCs). Circulating neutrophils can cluster around tumor cells and induce tumor cell aggregation, aiding tumor cell survival by hiding them from immune surveillance (53). Neutrophil extracellular traps (NETs) are webs of decondensed chromatin fibers conjugated together with histones, myeloperoxidase (MPO), elastase, and other cytoplasmic proteins (54). Recent studies also found that neutrophils could form many NETs both in circulation and in tumor lesions and could coordinate with platelets to capture CTCs and facilitate cancer metastasis (55). In addition, neutropenia is very common in cancer patients undergoing chemotherapy, and supportive treatment with granulocyte colony-stimulating factor (G-CSF) can induce a neutrophilic response; as a consequence, neutrophils are primed toward a pro-NETotic phenotype and may suppress the cytotoxic activity of T cells as well as impair immune surveillance (24, 56, 57). On the other hand, lymphocytes have the propensity to mount an adaptive antitumor response in malignant disease (58), and decreased lymphocyte numbers are considered to be related to an insufficient immunologic reaction, which may increase the risk of tumor relapse or metastasis (59). Clearly, a general association between prognosis and the NLR exists in BC, but large prospective studies and rigorous research are still required to determine its clinical significance.

## Mechanism of Neutrophil Chemotaxis to the Tumor Microenvironment

Neutrophils are considered the main immune cells that provide protection against invading pathogens, which can be induced by trauma, infection, and malignant disease (60). The recruitment of neutrophils is greatly dependent on certain chemokines, including interleukin (IL)-8 (also known as CXCL-8), CXCL-1, and CXCL-2 (61). IL-8 is a proinflammatory cytokine and acknowledged as the most important chemoattractant for neutrophils in the tumor microenvironment (62). IL-8 mainly comes from endothelial cells (ECs) and monocytes in the tumor microenvironment upon certain stimulation, such as physical injury, hypoxia, chemotherapy or radiotherapy, and other cell types, including fibroblasts and keratinocytes, can secrete IL-8 as well (63, 64). In addition to its chemotactic effect, it was revealed that IL-8 could provoke neutrophils to release NETs to assist cancer cell migration (5). By live-cell fluorescence microscopy, Gupta et al. confirmed that activated ECs could induce NETosis characterized by typical extracellular DNA lattices when cocultured with polymorphonuclear neutrophils (PMNs) and activated ECs (65). In addition, activated ECs produce other inflammatory cytokines, such as P-selectin, E-selectin, and intercellular adhesion molecule 1 (ICAM-1), to facilitate neutrophil adhesion to ECs and migration (66). Furthermore, tumor-promoting neutrophils in BC cells are also characterized by high expression of matrix metalloproteinases-9 (MMP-9) (67, 68), which was found to cleave CXCL-5, potentiating its action in neutrophil recruitment as a positive feedback function in tumors (15, 69). IL-17 was also found to control neutrophil recruitment in lung metastasis of BC in a mouse model: CD3^+^CD4^+^ and γδ T cells were the major sources of IL-17 (70, 71), and it was interesting to find that the absence of γδ T cells or neutrophils markedly reduced pulmonary and lymph node metastases without influencing primary tumor progression, which suggested a collaborative relationship between γδ T cells and neutrophils in promoting BC lung metastasis. However, in an orthotopic hepatocellular carcinoma model, Sofia et al. reported that TANs exert an overt antitumor role by suppressing γδ T17 cells via reactive oxygen species (ROS) (72), contrary to the phenomenon that within the 4T1-derived BC model, CD11b+/Ly-6G+ neutrophils that infiltrate and surround liver metastases were found to be tumor promoting (73). These controversial results suggest both promoting and suppressive roles of TANs in different circumstances.

High-mobility group box 1 (HMGB1) usually acts as a damage-associated molecular pattern that is released by dying cells or stressed cells to initiate inflammation and was later found to be an important chemoattractant for neutrophils (74). Epithelial cell-derived HMGB1 was found to recruit neutrophils to the necrotic site through its receptor RAGE (75). Enrichment of platelets has been reported in the microenvironment of multiple cancers, including BC (76), and infiltrating platelets could be activated by the large amounts of adenosine phosphate released by necrotic cells as a result of chemotherapy (77). Activated platelet-derived HMGB1, known as the major mediator of injury-induced thrombosis *in vivo* (74), can also stimulate NETosis through Toll-like receptor 4 (TLR4) and RAGE on neutrophils, and as a positive feedback mechanism, released NETs strongly induce a prothrombotic state and activate platelets (78). Meanwhile, tumor cell-derived exosomal HMGB1 was also found to activate neutrophils through the TLR4/NF-κB pathway, which promotes its survival by increasing the autophagic response and polarizing TANs to a protumor type (79). It is noteworthy that various reports imply the core position of the NF-κB pathway in the activation and recruitment of neutrophils (80, 81). In addition to HMGB1, tumor cells, including BC cells, have been reported to secrete other peptides, such as a2 isoform V-ATPase (a2V), to activate the NF-κB pathway in neutrophils, thereby promoting their recruitment and inhibiting their apoptosis (82, 83). Additionally, breast involution after weaning is characterized by acute inflammation and an increase in estrogen. It was found that estrogen could induce the mammary infiltration of neutrophils and upregulate the expression of protumor cytokines/chemokines, such as COX-2 and MMPs, in mammary infiltrating neutrophils (84).

In addition, similar to lymphocytes and macrophages, neutrophils are more likely to localize in tumors of triple-negative breast cancer (TNBC) than to tumors of other BC subtypes (85). Recently, Zhang et al. identified neutrophils and macrophages as the most frequent infiltrating immune cells in various BC murine models, and BC could be classified into a macrophage-enriched subtype (MES) and a neutrophil-enriched subtype (NES). It was interesting to find that there were only a few neutrophils in the MES but a large number of macrophages in the NES (57). This mutual repelling phenomenon in the MES and NES may result in spatial segregation within the same tumor. The authors speculated that a possible mechanism could be the factors derived from macrophages that inhibit the IL-8-dependent chemotaxis of neutrophils (86).

## Antitumor Function of TANs in BC

The polarization of neutrophils can be differentially regulated in the tumor microenvironment. In a mouse model, Fridlender et al. found that TANs from the early tumor stage were like tumor-killing cells, which produce high levels of hydrogen peroxide (H_2_O_2_), tumor necrosis factor (TNF)-α and NO, and that TANs are more likely to obtain a protumorigenic phenotype with tumor progression (87). Although few studies have directly compared the phenotype and function of TANs between early- and late-stage tumors, there are still some clues to support this hypothesis. A phenotypical and functional analysis of TANs in early-stage lung cancer found an activated phenotype (CD62^low^CD54^high^) that was able to stimulate T cell proliferation and IFN-γ release, which suggested a pro-inflammatory rather than immunosuppressive state of TANs in early-stage lung cancer (88). MPO is an enzyme characteristic of mature “N1” type neutrophils, which are able to convert H_2_O_2_ to cytotoxic hypochlorous acid (HOCI) (87, 89). Recently, a retrospective study of 928 BC cases revealed that MPO-positive neutrophils (defined as ≥5 cells/tissue punch) were found in 16% of evaluable cases, while the luminal (ER/PR+ and Her2-), Her2-enriched and triple-negative types had positive rates of 13, 29.7, and 26.4%, respectively, in addition, in univariate analyses, infiltration by MPO-positive neutrophils was a significant independent favorable indicator for both OS and DFS. Notably, almost all of the patients included in this study had early-stage disease (T1-2 72%, N0-1 89%), and the data suggested that MPO-positive neutrophils were much more abundant in BC cases with low T and N stages than in advanced cases (90).

In addition, a direct tumor killing function of neutrophils has also been reported. One of the classical factors working against tumor cells is ROS. Recent research in mouse BC models revealed that ROS-mediated cell lysis was dependent on Ca^2+^ channels and mediated by transient receptor potential cation channel, subfamily M, member 2 (TRPM2) expression on tumor cells (91). Although TCGA analysis revealed a high expression of TRPM2 in BC cells (http://gepia2.cancer-pku.cn/#index), active NOX1, catalase and SOD were also increased in the membrane of cancer cells, forming a complex mechanism by which tumor cell apoptosis induced by ROS is prevented (92). In addition, tumor cells are characterized by enhanced metabolic activity and high levels of intracellular ROS (93), which indicates that direct cytotoxic effects of neutrophil-produced ROS are not sufficient. In addition to the direct cytotoxic effect, TANs containing ROS have been found to strongly suppress IL-17-producing γδ T cells (72), which are critical for shaping the immune suppressive microenvironment in various solid tumors (94–96), and have also been reported to promote BC cell extravasation and metastasis (71). In addition, neutrophils could also express Fc receptors and exert antibody-dependent cellular cytotoxicity (ADCC) effects similar to those of T cells and macrophages, leading to a trogocytosis effect to destroy cancer cells (97). However, some studies have indicated that neutrophils are more likely to be distributed at the periphery of tumors at the initiation stage (85, 87), which may make controlling tumor growth with these cell-cell contact-dependent mechanisms ineffective.

## Protumor Effects of TANs

More studies suggest that neutrophils facilitate tumor promotion and metastasis in BC than antitumor effect. Overexpression of the chemokines CCL2 and CCL17 is a recognized feature of N2 neutrophils. Richmond et al. (98) found that exogenous CCL2 enhances the killing effect of neutrophils against BC cells *in vitro*, while this antitumor activity was not observed *in vivo*. Instead, intranasal delivery of CCL2 to BALB/c mice markedly enhanced lung metastasis of BC cells and increased the recruitment of CD4+ T cells and CD8+ central memory T cells. CCL17 secretion from TANs was found to support tumor growth by recruiting CD4+ Treg cells and macrophages (99). In addition to recruiting immune-suppressive cells, TANs were reported to promote the accumulation of BC cells in the lung and directly inhibit natural killer (NK) cell-mediated clearance of tumor cells (100). Human NK cells can be divided into CD56^dim^ (antitumor) and CD56^bright^ (protumor) subsets, and CD56^bright^ NK cells are enriched in the tumor microenvironment and draining lymph nodes (101, 102). Early reports revealed that ROS and arginase-1 from neutrophils impair the maturation and cytotoxic function of NK cells (103), but CD56^bright^CD16^−^ NK cell are resistant to neutrophil-derived ROS, perhaps due to their high antioxidative capacity (104). Meanwhile, NK cells could be recruited by TANs via CCL2 and CCL5, which may explain the preferential accumulation of CD56^bright^ NK cells in tumor microenvironments with high ROS levels (105).

Extracellular arginine is crucial to signal local CD8+ cells and increase their CD3ζ expression, which is key for T cells to survey antigens presented on MHC class I molecules, and it was also found to be necessary for T cell activation and survival (106). Tumor cell-derived IL-8 could lead to TAN degranulation, resulting in arginase-1 release and conversion of extracellular arginine to ornithine and urea, thereby dampening the survival and cytotoxic effect of CD8+ T cells (53, 107, 108). Neutrophil elastase (NE) is also released by TANs and can be endocytosed by tumor cells via neuropilin-1 (NRP1); this results in the cross-presentation of PR1, which is an NE-derived HLA-A2-restricted peptide that may be an immunotherapeutic target (109). Besides, upon endocytosis, NE is to bind insulin receptor substrate-1 (IRS-1), which removes the inhibitory effect of IRS-1 on phosphatidylinositol 3-kinase (PI3K) to enhance the proliferation of cancer cells (110).

Recent reports highlighted the leukocytes, especially neutrophils preferentially uptake tumor derived extracellular vesicles, or named exosomes (111). Hypercoagulability is one of the important characteristics of malignant tumors, and has been reported associated with NETs. Breast cancer cell 4T1-derived exosomes induced NETs formation in neutrophils, besides, tumor-derived exosomes also interacted with NETs to significantly accelerate venous thrombosis *in vivo* (112). Furthermore, several reports also indicated the cancer derived exosomes prolonged lifespan of neutrophils, and also polarized neutrophils toward pro-tumor type (79, 113).

In addition to direct modulation of the protumor microenvironment, increasing evidence has found that neutrophils promote tumor cell migration and the formation of a metastatic niche (6, 13, 114). Tumor angiogenesis is regarded as a prerequisite for tumor metastasis, and TANs have been recognized as an important source of vascular endothelial growth factor (VEGF) upon specific stimulation in the tumor microenvironment (115, 116). Neutrophils were also found to be one of the main sources of MMP-9 (117), and the link between MMP-9 and VEGF has been reported previously. The absence of MMP-9 has been reported to have a similar function as the inhibition of VEGF signaling, indicating that MMP-9 serves as an angiogenic switch during tumorigenesis by inducing VEGF release from the matrix (117–119). In addition, Gabriele et al. also found that MMP-9 was expressed by a small number of cells in close proximity to the vasculature, such as infiltrating inflammatory cells, rather than tumor cells (118). In addition, several serine proteases are also produced by TANs, such as NE, cathepsin G and proteinase-3, which have been reported to activate MMP-2 to promote tumor invasion and proliferation (120, 121). In addition, although neutrophils were reported to produce little tissue inhibitor of matrix metalloprotease (TIMP-1), Wang et al. observed that BC cells with CD90-positive expression could induce the TIMP-1 secretion by TANs, and as a reciprocal effect, TIMP-1 induced EMT and metastasis in BC (122). Other neutrophil-derived cytokines such as IL-1β, IL-6, and IL-17α have been reported to initiate EMT of cancer cells by activating JAK2/STAT3 and ERK signaling (123, 124).

In addition to modulating the primary tumor microenvironment, neutrophils can also assist the formation of the cancer premetastatic niche in distant organs. CTCs are precursors for metastatic lesion formation; intravascular NETs were found to protect CTCs from attack by circulating immune cells; and dysregulated NETs were found to induce inflammatory vascular injury, EC shrinkage and tissue damage (53, 125–127). Moreover, *in vitro* and *in vivo* experiments found that activated neutrophils promote the adherence of CTCs to ECs and facilitate their lung and liver metastasis (128). Recently, Aceto et al. provided strong evidence that neutrophils escort CTCs in BC to assist metastasis (129). With detection of cell surface markers and Wright Giemsa staining, they identified that most CTC-associated white blood cells were N2-like neutrophils. In addition, single-cell RNA sequencing revealed higher Ki-67 expression in disseminated tumor cells from CTC neutrophil clusters than in standalone CTCs. In the same study, TNF-α, oncostatin M, IL-1β, and IL-6 were frequently expressed by CTC-associated neutrophils and matched by the receptors on corresponding CTCs; on the other hand, CTCs from the CTC neutrophil clusters expressed high gene levels encoding G-CSF, transforming growth factor (TGF)-β3 and IL-15, which have been reported to activate neutrophils (130–132), illuminating a mechanism of neutrophil-CTC cluster formation.

In addition to escorting CTCs in circulation, several studies have found that neutrophil accumulation is a prerequisite for cancer metastasis. For both orthotopic transplantation and spontaneous BC models, neutrophils were suggested to accumulate in the distant organ before cancer cells infiltration (6, 133). Obesity and elevated cholesterol are risk factors for BC development and poor prognosis (134, 135). Interestingly, 27-hydroxycholesterol (27HC) increased the number of polymorphonuclear-neutrophils and γδ T cells at distal metastatic sites, and neutrophils were required for the metastatic effects of 27HC (136). Egeblad et al. (137) developed a confocal intravital lung imaging system and found that NETs were formed early in the lung and continued to form for the next few days after tail vein injection of BC cells. In addition, based on immunofluorescence staining of human primary BC and matched metastatic lung lesions, they found that the abundance of NETs was highest in TNBC, but NETs were absent or very rare in luminal BC samples, which may explain the higher metastatic ability of TNBCs than luminal BCs. In ovarian cancer, an influx of neutrophils in the omentum was also observed before metastasis, and blockade of NET formation with peptidyl arginine deiminase 4 (PAD4), an enzyme that is essential for NET formation, could decease omental colonization of cancer cells (133). In addition to supporting colonization of cancer cells, lung-infiltrating neutrophils has also been reported to directly promote cancer proliferation via release of high levels of S100A8, S100A9, Bv8, MMP-9 and the lipid leukotriene B4, which stimulate the migration and proliferation of BC cells, and activate the MAPK/Erk pathway in BC cells to potentiate their tumorigenic capacity (6, 138). Interestingly, BC can remain dormant and clinically undetectable before late recurrence decades later, and it has been reported that inflammation induced by stimuli such as lipopolysaccharide or smoking triggers neutrophils to accumulate and NET formation, which can cause tumor recurrence by activating the integrin and FAK/ERK/MLCK/YAP signaling pathways to awaken dormant tumor cells (139). Overall, evidence is mounting that neutrophils play a significant detrimental role in every step of cancer metastasis.

## Spatial Distribution and Various Cluster of TANs

**Figure 1:**
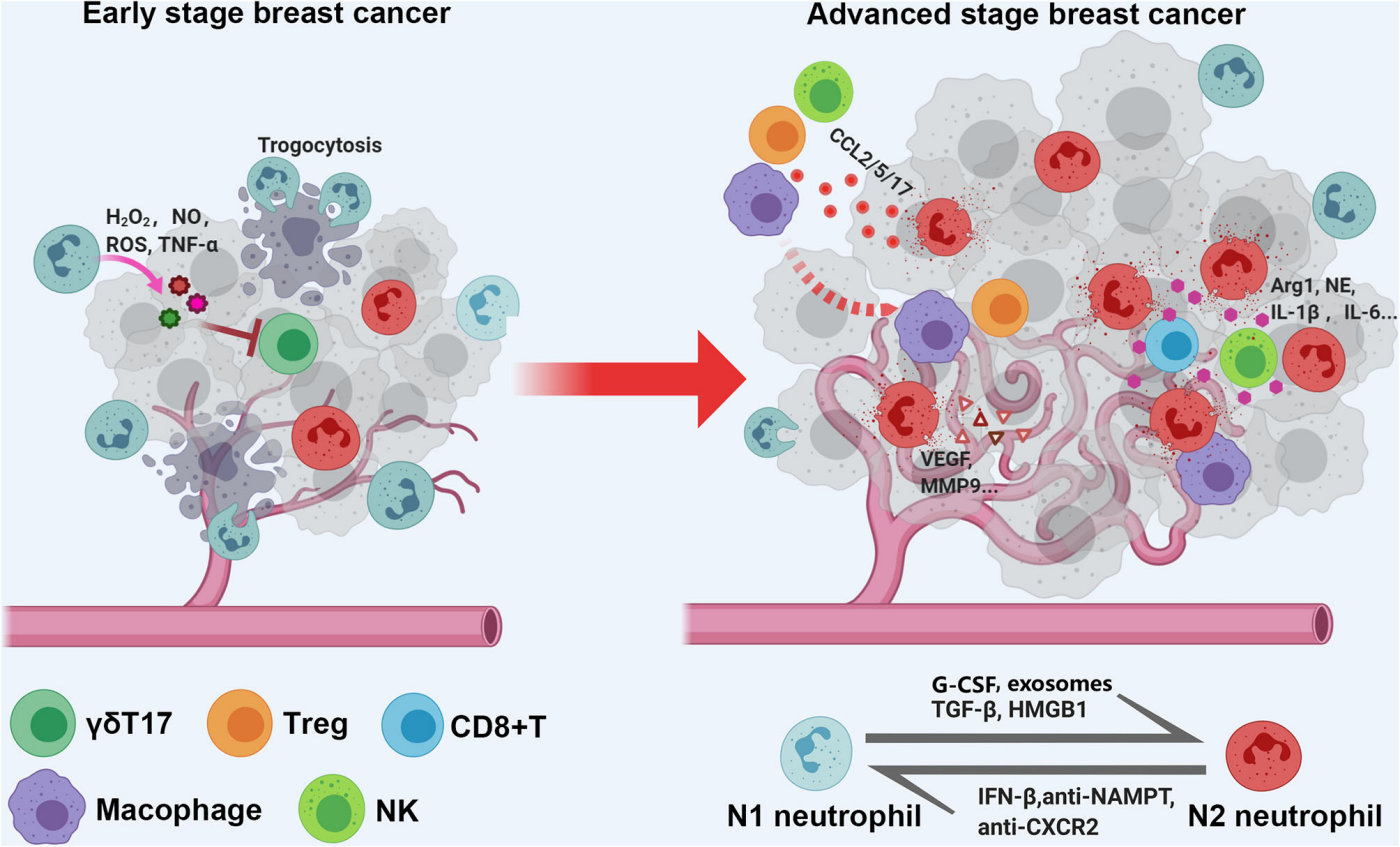
Schematic diagram of spatial distribution and functional of TANs in early and late stage breast cancer. As a quick response, tumor associated neutrophils (TANs) are scattered around the periphery of the tumor site in early stage, and exert tumor inhibition function; with tumor progression, the TANs are more likely to distributed among and direct contact with tumor cells, and function as tumor promoting cells via shaping immune suppressive microenvironment, enhancing angiogenesis, and caner metastasis.

Several studies have suggested that the spatial distribution of TANs is different between early- and advanced-stage cancers, which is related to the biological function of TANs (antitumor or protumor functions) (Figure 1). A mouse model of lung carcinoma and mesothelioma revealed that TANs were scattered around the periphery of the tumor site in the early stage, while neutrophils were more distributed among the tumor cells in the late stage (87). Another retrospective study of BC defined TANs as neutrophils in direct contact with carcinoma cells and showed that 47.7% of cases were TAN positive, but the frequency of cancer cell contacting-TANs was much higher in advanced-stage cases than in early-stage cases (85), which also indicates that neutrophils are dynamically modulated by the tumor microenvironment both in phenotype and spatial position. Recently, Wang et al. evaluated the association between parenchymal and stromal neutrophil counts and clinical outcomes with their own BC datasets and found that neutrophils in the tumor parenchyma, rather than those in the stroma, were an independent poor prognostic factor (122).

**Figure 2:**
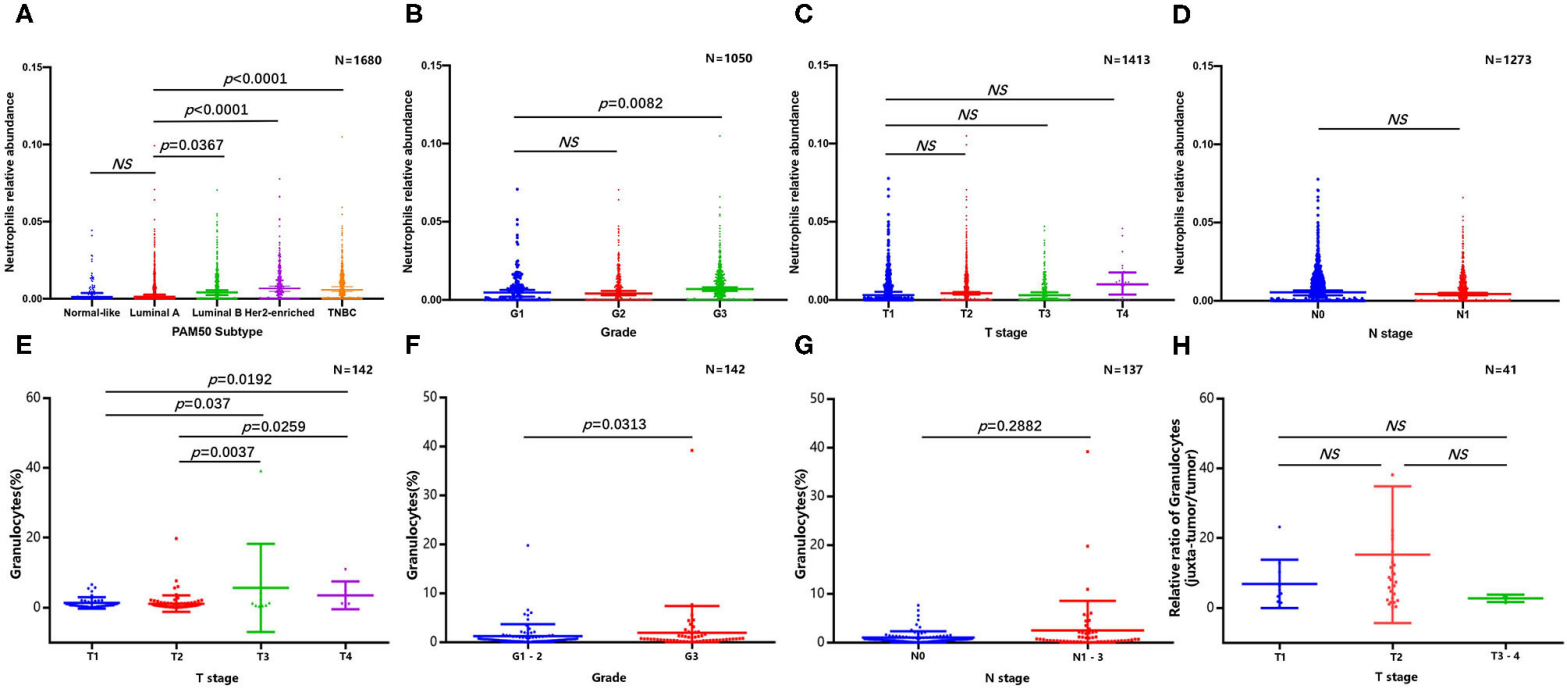
Abundance of tumor infiltrating neutrophils in breast cancer. CIBERSORT algorithm analysis was performed based on mRNA expression datasets (GSE6532, GSE9195, GSE16446, GSE17907, GSE19615, GSE20685, GSE20713, GSE21563, GSE31448, GSE42568, GSE48390, and GSE58984). Comparison of breast cancer infiltrating neutrophils with different molecular subtype **(A)**, grade **(B)**, tumor size **(C)**, and lymph nodes metastasis **(D)**; original data based on mass cytometry (141) was extracted and re-analyzed to evaluate the abundance of neutrophil stratified with different tumor size **(E)**, grade **(F)**, and lymph nodes metastasis **(G)**, relative proportion of neutrophils between juxta-tumoral and tumor tissues among different tumor size was also presented **(H)**.

Because of the lack of direct information from previous publications, we tried to determine the abundance and subtype of TANs in human BC via the CIBERSORT-LM7 deconvolution algorithm based on mRNA expression datasets (GSE6532, GSE9195, GSE16446, GSE17907, GSE19615, GSE20685, GSE20713, GSE21563, GSE31448, GSE42568, GSE48390, and GSE58984) (140). Our analysis indicated that the proportion of neutrophils was significantly higher in BC cases with a higher grade and of the luminal B, TNBC and HER2+ subtypes but was not associated with tumor size or axillary lymph node metastasis (Figures 2A–D). Recently, Klein et al. (142) used single-cell RNA sequencing (scRNA-seq) to map tumor-infiltrating myeloid cells in non-small-cell lung cancer patients and revealed that tumor-infiltrating neutrophils (TINs) could be clustered into five subsets (hN1-hN5). hN1 cells were characterized by high expression of Arginase-1, MMP9/8, S100A8 and S100A9, and ADAM8. As we discussed in the previous section, almost of these genes play a tumor-promoting role in BC. Another earlier research focused on immune microenvironment also profiled BC infiltrating 45,000 immune cells with scRNA-seq, and identified neutrophils in half of the patients. However, the neutrophils and mast cells were excluded in analysis due to their great heterogeneity (143). In addition, Wagner et al. (141) performed a single-cell analysis to map the microenvironment of BC using mass cytometry, and found the abundance of neutrophils (also termed as granulocytes) significant higher in juxta-tumoral tissue than tumor, and it is noted that nearly 90% of the included patients were early stage (IA-IIB) and luminal subtype. Since the CIBERSORT and scRNA-seq analysis are both based on transcriptome level, here we extracted the original data of Wagner's study to evaluate the neutrophil distribution stratified with different pathological features again (141). The results confirmed the frequency of TANs were greater in tumor with larger size and higher grade, but not associated with lymph nodes metastasis (Figures 2E–H); besides, we also compared the relative proportion of neutrophils between juxta-tumoral and tumor tissues among different tumor size, the negative results (Figure 2H) suggested that the increase of neutrophils infiltration in tumor may be a continuous chemotactic process from para-tumoral tissue toward the tumor. More rigorous experiments are needed in the future to delineate the dynamic changes in neutrophil function during this process.

## The Impact of Chemotherapy and Radiotherapy on TANs

Chemotherapy and radiotherapy are integral parts of BC treatment that can influence the immune microenvironment. Anthracycline and cyclophosphamide-based chemotherapy regimens are still widely used in BC treatment (144, 145). It has been reported that anthracycline as well as cyclophosphamide can impair the actin polymerization of neutrophils, which results in insensitivity of neutrophils to the chemotactic effect of IL-8, therefore decreasing the infiltration of neutrophils in BC (146). In addition, the migration ability of neutrophils was also impaired by paclitaxel, a cornerstone drug in BC treatment, which could be attributed to the increased cell stiffness and decreased compliance induced by enhanced microtubule assembly (147). Platinum-based chemotherapeutic strategies have also been widely applied in various solid malignancies, such as colorectal cancer, ovarian cancer, and BC (148–150). Determination of neutrophil-specific chemokine expression by RT-PCR confirmed that oxaliplatin plus lipid A, which has been reported to exert antitumor effects against different tumor types, including colon cancer, BC and melanoma (151, 152), increased CXCL-1, CXCL-2, and IL-8 gene expression in tumors, thereby stimulating recruitment of antitumor N1-like neutrophils and impeding cancer progression (153).

The impact of radiotherapy on neutrophils has also been reported. In the EMT6.5 mammary tumor model, conventional radiotherapy (CRT) but not microbeam radiation therapy (MRT) induced a substantial increase in TAMs and TANs, and increased levels of CCL2 (which, as mentioned above, can be released by TANs to exert chemoattractant functions) were also observed in tumors subjected to CRT (154). In addition, different radiation regimens (20 Gy, 4 × 2 Gy, 2 Gy, or 0 Gy) induce different immune responses. High single doses (20 Gy) induce a delayed type of primary necrosis with characteristics of mitotic catastrophe and plasma membrane disintegration. The protein damage-associated molecular patterns (DAMPs) released by these dying cells stimulate sequential recruitment of neutrophils and monocytes *in vivo* (155). Furthermore, elevated infiltration of neutrophils was observed in various radiation-induced pneumonia models (156–158). However, in human BC, there is no direct evidence for how radiation affects the variations in neutrophils in the tumor microenvironment, but it has been reported that the NLR could be an independent prognostic factor in TNBC following radiotherapy (159).

## Targeting Neutrophils for Cancer Treatment

Since the majority of studies have revealed a protumor function of neutrophils in BC, targeting neutrophils as a therapeutic strategy has been investigated. In mouse mammary tumor models, depletion of neutrophils with anti-Ly6G antibodies resulted in diminished tumor formation and lung metastasis (160). In addition, multiple BC xenograft models have proved that tumors enriched in neutrophils are more likely to be resistant to immune checkpoint blockade (ICB) therapy, while depleting neutrophils could restore the efficiency of ICB to reduce tumor recurrence and significantly improve progression-free survival (57). However, it is impossible to eradicate all neutrophils in cancer patients since it would cause severe immunodeficiency and infection, so it is more desirable to block the chemotaxis of neutrophils in tumor tissues or to prevent their polarization to the N2-like phenotype.

IFN-β and TGF-β are the cytokines that are most often reported to modulate the switch between N1 and N2-like neutrophil polarization. Steven et al. first revealed that TGF-β blockade significantly increased the influx of antitumor neutrophils and activated CD8+ T cells in a BC mouse model (161). Thereafter, a population of low-density neutrophils (LDNs) featuring impaired antitumor function and immunosuppressive properties accumulated continuously with cancer progression, including in BC. This LDN subpopulation consists of both immature MDSCs and mature neutrophils that are transformed from “normal,” antitumor, high-density neutrophils (HDNs) in a TGF-β-dependent mechanism (162). Mice deficient in IFN-β showed rapid tumor growth and large amounts of neutrophil infiltration with high expression of c-myc and stat3, which are known as enhancers of MMP-9, VEGF, and CXCR4 expression (163). Furthermore, nicotinamide phosphoribosyl transferase (NAMPT), an enzyme with cytokine-like features involved in the salvage pathway of nicotinamide adenine dinucleotide (NAD) biosynthesis (164), was found to be highly expressed and to modulate the tumorigenicity of TANs. Targeting NAMPT in TANs led to their antitumor conversion and antiangiogenic polarization by inhibiting SIRT1 signaling, which resulted in efficient repression of tumor growth (165). In addition, CXCR2 blockade in a K-ras mutant mouse model of lung cancer induced tumor regression, which was related to reduced neutrophil chemotaxis and polarization from N2- to N1-like cells (166). Transfusion of neutrophils (granulocyte transfusion, GTX) to cancer patients has also been tested, but due to the short life span of neutrophils and severe adverse events, such as respiratory distress and even death, it needs further investigation (167).

## Conclusions

**Figure 3:**
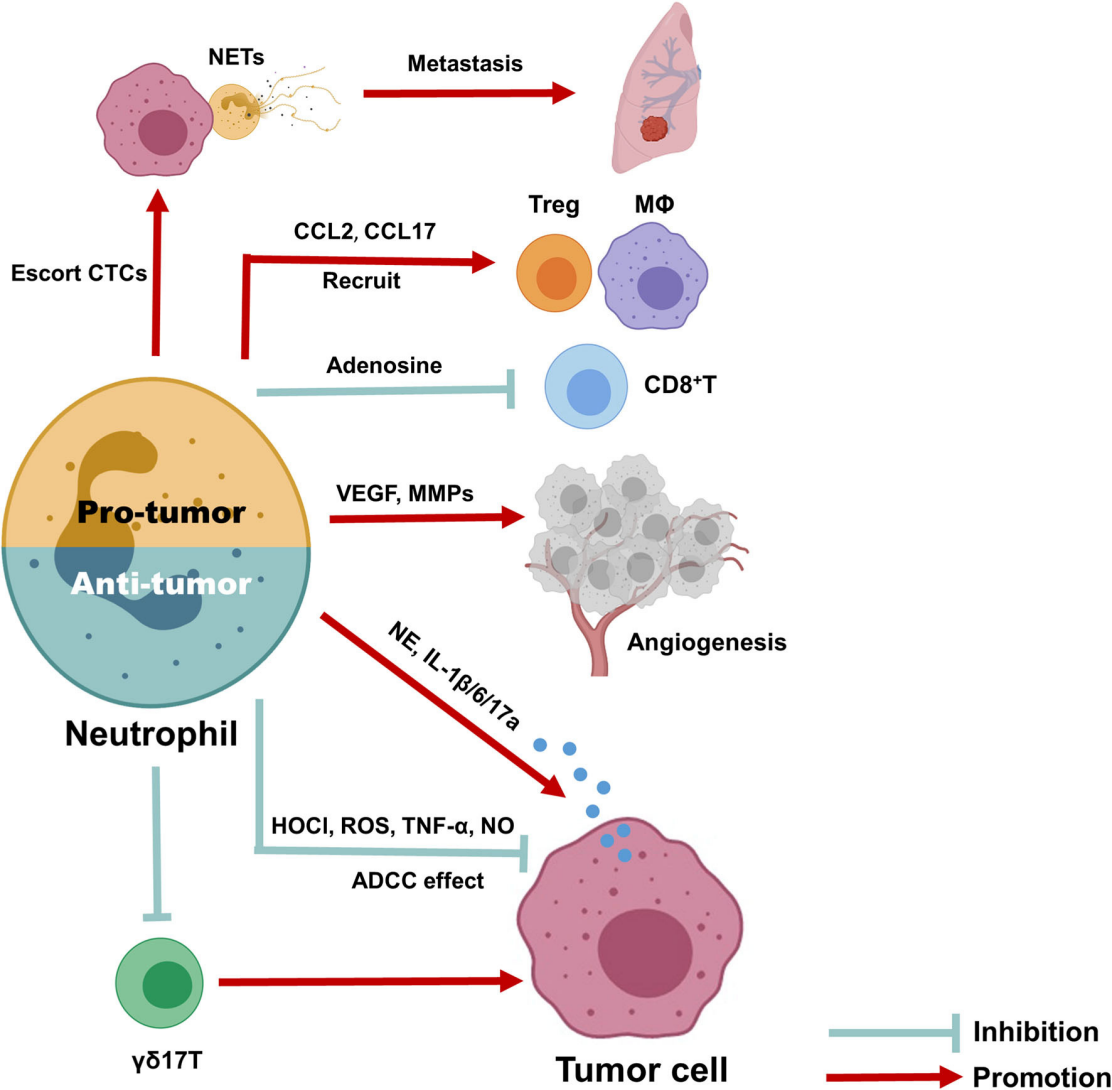
Anti- and pro-tumor function of neutrophils in breast cancer. Due to the dynamic regulation of neutrophils in tumor microenvironment, it can either function as inhibit or promote tumor progression. The anti-tumor neutrophils can exert anti-tumor function through antibody-dependent cellular cytotoxicity (ADCC) effect, produce HOCI, ROS, TNF-α, and NO as direct killing effect, and suppress immune suppressive cells, such as IL-17 producing γδ T cells. To the contrary, pro-tumor neutrophils can produce CCL2 and CCL17 to recruit CD4+ Treg cells and anti-inflammatory macrophages, together with release arginase-1 to inhibit the activation of CD8+ cells, therefore promote immune suppressive microenvironment; they also promote tumor angiogenesis via release MMP9 and VEGF and produce NETs to escort circulating tumor cells and promote cancer metastasis; finally, neutrophils could release elastase, IL-6, IL-1β, and IL-17 to promote tumor cells proliferation and EMT directly.

A schematic picture depicting the dual role of neutrophils in BC (Figure 3). Here, we provide a comprehensive review of circulating and TINs in BC to highlight their importance in the tumor microenvironment. Although increasing evidence suggests a close association of neutrophils with treatment outcome and prognosis in BC, as well as their utility in predicting these parameters, it is still difficult to utilize the NLR or TANs as clinical tools due to the lack of reliable markers to distinguish N1 and N2 neutrophils and the lack of a unanimous cut-off value for the NLR. In addition, existing evidence suggests an interesting phenomenon in which the spatial distribution and function of neutrophils are dynamically regulated with tumor progression, although the detailed mechanism requires further research. Overall, exploring more effective and low-toxicity strategies to inhibit protumor neutrophil polarization is a promising approach for cancer treatment.

## Author Contributions

YS and CN wrote the manuscript. CZ and WZ edited the manuscript. YS, WZ, and SP collected the related literatures. HH, JJ, and WC finished the tables and figures. TZ provided the feedback and guidance. All authors read and approved the final manuscript.

## Conflict of Interest

The authors declare that the research was conducted in the absence of any commercial or financial relationships that could be construed as a potential conflict of interest.

## Abbreviations

BC: Breast cancer
MDSCs: Myeloid-derived suppressor cells
TANs: Tumor-associated neutrophils
NLR: Neutrophil-to-lymphocyte ratio
DFS: Disease-free survival
OS: Overall survival
ALC: Absolute lymphocyte count
NCT: Neoadjuvant chemotherapy
pCR: Pathological complete response
PLR: Platelet-to-lymphocyte ratio
TAMs: Tumor-associated macrophages
CTCs: Circulating tumor cells
NETs: Neutrophil extracellular traps
MPO: Myeloperoxidase
G-CSF: Granulocyte colony-stimulating factor
ECs: Endothelial cells
PMNs: Polymorphonuclear neutrophils
ICAM-1: Intercellular adhesion molecule 1
MMP-9: Matrix metalloproteinases-9
ROS: Reactive oxygen species
HMGB1: High-mobility group box 1
TLR4: Toll-like receptor 4
TNBC: Triple-negative breast cancer
MES: Macrophage-enriched subtype
NES: Neutrophil-enriched subtype
H_2_O_2_: Hydrogen peroxide
TNF-α: Tumor necrosis factor-α
HOCI: Hypochlorous acid
TRPM2, Transient receptor potential cation channel, subfamily M: member 2
ADCC: Antibody-dependent cellular cytotoxicity
NK: Natural killer
NE: Neutrophil elastase
NRP1: Neuropilin-1
IRS-1: Insulin receptor substrate-1
PI3K: Phosphatidylinositol 3-kinase
VEGF: Vascular endothelial growth factor
TIMP-1: Tissue inhibitor of matrix metalloprotease
TGF-β: Transforming growth factor-β
27HC: 27-hydroxycholesterol
PAD4: Peptidyl arginine deiminase 4
TINs: Tumor-infiltrating neutrophils
CRT: Conventional radiotherapy
MRT: Microbeam radiation therapy
DAMPs: Damage-associated molecular patterns
ICB: Immune checkpoint blockade
LDNs: Low-density neutrophils
HDNs: High-density neutrophils
NAMPT: Nicotinamide phosphoribosyl transferase
NAD: Nicotinamide adenine dinucleotide
GTX: granulocyte transfusion.
